# Histopathological Study of Chronic Hepatitis B: A Comparative Study of Ishak and METAVIR Scoring Systems

**Published:** 2010-11-01

**Authors:** M. Mohamadnejad, S. M. Tavangar, M. Sotoudeh, F. Kosari, M. Khosravi, B. Geramizadeh, G. Montazeri, A. Estakhri, M. M. Mirnasseri, A. Fazlollahi, F. Zamani, R. Malekzadeh

**Affiliations:** 1 *Digestive Disease Research Center, Tehran University of Medical Sciences, Tehran, Iran, *; 2 *Gastrointestinal and Liver Disease Research Center, Iran University of Medical Sciences, *; 3 *Departments of Pathology, Shariati Hospital, Tehran University of Medical Sciences, Tehran, *; 4 *Sina Hospital, School of Medicine, Tehran University of Medical Sciences, Tehran, Iran, *; 5 *Organ Transplant Research Center, Nemazi Hospital, Shiraz University of Medical Sciences, Shiraz, Iran*

**Keywords:** Hepatitis B, Chronic hepatitis, Staging, Ishak, METAVIR

## Abstract

Background: Ishak and METAVIR scoring systems are among the most commonly used histopathological systems to evaluate chronic hepatitis.

Objective: To assess the level of agreement between these two scoring systems in patients with chronic hepatitis B.

Methods: Liver biopsy samples taken from 92 patients with chronic hepatitis B were considered as the training set; 57 more biopsy specimens were used as the validation set. In the training set, grade of necroinflammation and stage of fibrosis for each liver biopsy specimen were determined by two expert liver pathologists using both Ishak and METAVIR systems. Inter-observer variability between the two pathologists was evaluated. Biopsy specimens of the validation set were seen and scored by a third expert pathologist. In the training set, criteria were developed to categorize Ishak grading and staging systems separately to best fit with the METAVIR scoring system. The criteria found in the training set, was then tested in the validation set. The level of agreement between the two scoring systems was assessed by weighted kappa statistics.

Results: For the training set, agreement between the two pathologists was excellent. Using our proposed criteria in the training set, there was excellent level of agreement in grading (κ = 0.89) and staging (κ = 0.99) between Ishak and METAVIR systems. In the validation set, the criteria led to substantial correlation (κ = 0.61) in grading, and excellent correlation (κ = 0.94) in staging between the two systems.

Conclusion: Using our proposed criteria, excellent or at least substantial concordance between Ishak and METAVIR scoring systems can be achieved for the degree of both necro-inflammatory changes and fibrosis.

## INTRODUCTION

Liver biopsy is considered the gold standard for assessing the grade of liver injury and stage of liver fibrosis in patients with chronic hepatitis. In attempt to standardize assessment of liver histology by pathologists, several scoring systems have been developed. Among these, modified Histology Activity Index (HAI) developed by Ishak, et al [1], and the METAVIR system [2,3] are used most widely. While a large number of researchers use Ishak system to assess liver histology in chronic hepatitis studies [4,5], other researchers—mostly from Europe—prefer the METAVIR system [6]. 

Each of these scoring systems provide reliable scores, with relatively little intra- and inter-observer variations [3,7]. In a recent study, a good concordance between Ishak and METAVIR systems was reported [8], though variation was greater for necro-inflammatory features than for fibrosis and cirrhosis. 

It is, however, unclear whether a given score in the Ishak system predictably corresponds to a certain score in the METAVIR system. Concordance of the two systems in the grading of necro-inflammatory changes is more problematic. It is not known if individual components of the grading scores in the Ishak system (*e.g.*, interface hepatitis, confluent necrosis, *etc*) contribute to this correlation. We, therefore, attempted to identify criteria in the Ishak system which corresponds to the METAVIR score. 

## MATERIALS AND METHODS

One hundred and sixty eight consecutive liver biopsies from treatment naïve chronic hepatitis B virus (HBV) carriers sent to the Department of Pathology of our center between 2004 and 2005 were prospectively evaluated. All patients were chronic carriers of HBV documented with two positive HBs Ag tests, at least six months apart. Informed written consent for the study was given by each patient prior to liver biopsy.

Biopsy samples received between January 2004 and March 2005 were considered as training set, and the samples received between April 2005 and December 2005 were considered as validation set. 

Nineteen out of the 168 samples were excluded because of inadequate size of the specimens (*e.g.*, less than four portal tracts). Thus, 92 specimens were included as the training set, and 57 as the validation set. All specimens were fixed in 10% formalin, and embedded in paraffin. For each case, three sections were stained by hematoxylin-eosin, mason-trichrome, and reticulin. All biopsy specimens in the training group were seen by two pathologists expert in liver pathology—they had at least 10 years experience of practice in the field of liver pathology in an academic center. In the training set, all slides were reviewed by each pathologist and were scored by the Ishak system [1]. Subsequently, all specimens were scored by METAVIR system [2,3]. Each pathologist worked independently and was blinded to the results of the readings of the other colleague and the readings by the other system. Inter-observer agreement was evaluated by kappa statistics. Then, all discordances between the two pathologists were resolved by agreement in joint sessions. 

In order to compare the two scoring systems, we tried to equalize the number of categories in the two systems. Grading of necro-inflammation has four components in the Ishak system and includes “a”: interface hepatitis; “b”: confluent necrosis; “c”: focal lytic necrosis; and “d”: portal inflammation [1]. Grading of the METAVIR system is simply classified as A0 to A3 based on the severity of the necro-inflammation [3]. 

We pooled the grading scores of the Ishak system into four groups (*i.e.*, minimal, mild, moderate, and severe necro-inflammation). We tried to modify individual components of Ishak grading system in each group to find the categorization which best fits the METAVIR grading system (Table 1). These groups were compared with the four groups of METAVIR grading system by kappa statistics. 

**Table1:** Proposed criteria for comparison between Ishak and METAVIR scoring systems

Grading of necro-inflammation in the Ishak system*		Grading of necro-inflammation in the METAVIR system
Minimal	a0, b0, c0-1, d0-1	A0
Mild†	a0,b0, c ≥ 2 or and/or d ≥ 2	A1
	a1-2, b0, any c, any d	
	a0, b ≥ 1, any c, any d	
Moderate	a1-2, b ≥ 1, any c, any d	A2
	a 3-4, b0, any c, any d	
Severe	a3-4, b ≥ 1, any c, any d	A3

* In Ishak system letter “a” denotes interface hepatitis (piecemeal necrosis); “b” confluent necrosis; “c” focal lytic necrosis; and “d” portal inflammation.

† Either of these three different conditions are considered as mild necro-inflammation.

Additionally, we compared grading of METAVIR with the previously mentioned categories of the Ishak system, *i.e.*, minimal necro-inflammation (total grades of 1–3), mild (total grades of 4–8), moderate (total grades of 9–12) or severe (total grades of 13–18) [8]. 

The Ishak system scores fibrosis into seven categories (0–6), while the METAVIR system scores liver fibrosis into five groups (F0–F4). We modified the Ishak fibrosis scoring system by reducing the seven categories to five, and found the categorization which best fits the five groups of the METAVIR system in the training set (Table 2).

**Table 2:** Proposed criteria for comparison between Ishak and METAVIR scoring systems

Staging of liver fibrosis	
Ishak system	METAVIR system
0	F0
1 or 2	F1
3	F2
4 or 5	F3
6	F4

Then, the 57 biopsy specimens of the validation set were evaluated by a third expert pathologist. He first scored all the slides by the Ishak system. He then scored the slides by the METAVIR system. Categorization of the Ishak grading (Table 1) as well as Ishak staging (Table 2) obtained from the training set, was applied for the validation set. 

The same statistical analysis was done to compare Ishak and METAVIR systems in the validation group.

Statistical analysis

Weighted kappa statistics were used to determine the level of agreement in each analysis. 

A κ < 0.2 was considered as “slight” concordance; 0.2–0.39 considered “fair,” 0.4–0.59 “moderate,” 0.6–0.79 as substantial, and κ ≥ 0.8 was considered “excellent” or “almost perfect” level of concordance. 

## RESULTS

In the training set, 67 (73%) patients were male, and 25 (27%) were female. The mean±SD age of patients in the training set was 38.5±12.0 years. In the validation set, 35 (61%) patients were male, and 22 (39%) were female. The mean±SD age of patients in the validation set was 35.5±10.7 years. The biopsy specimens included a median number of seven (range: 4–22) portal tracts.

Inter-observer variability in the training set

In general, agreement between the two pathologists was excellent. In the Ishak system, the κ statistics of inter-observer agreement was 0.90 for interface hepatitis, 0.92 for confluent necrosis, 0.80 for focal necrosis, 0.87 for portal inflammation, and 0.86 for staging of fibrosis. In the METAVIR system, the κ of inter-observer agreement was 0.81 for grading of necro-inflammation, and 0.82 for staging of fibrosis.

Correlation between the two systems in the training set

Using the Ishak system, the mean±SD stage of liver fibrosis was 1.30±1.55 (range: 0–6). The mean±SD grade of necro-inflammation was 4.89±3.01 (range: 1–15) in the training set. Evaluation of the different components of the Ishak grading system showed that the mean±SD score of interface hepatitis was 1.18±1.10 (range: 0–4); the mean±SD score of confluent necrosis was 0.63±1.03 (range: 0–5); the mean±SD score of focal lytic necrosis-apoptosis was 1.52±0.54 (range: 0–3); and the mean±SD score of portal inflammation was 1.55±0.89 (range: 0–4). 

We categorized the Ishak scores of the training set (Table 1), and compared the groups by the METAVIR grading system. Table 3 shows a comparison of the necro-inflammatory scores between the two systems according to our proposed criteria. There was an excellent correlation (κ = 0.89) between the two systems using the proposed criteria. 

**Table 3:** Comparison between METAVIR grading system, and the proposed criteria for Ishak grading categorization in the training set*

METAVIR grading	Ishak grading			
	Minimal	Mild	Moderate	Severe
A0	22	0	0	0
A1	0	33	4	0
A2	0	3	17	0
A3	0	0	0	13
Total	22	36	21	13

* κ = 0.89

However, when we compared grading of METAVIR with the previously suggested categories of the Ishak grading system [8], we found a much weaker correlation between the two systems (κ = 0.18). Using our criteria, the correlation between staging of METAVIR and staging of Ishak system was excellent (κ = 0.99) (Table 4). 

**Table 4:** Comparison between scores for liver fibrosis obtained by METAVIR and Ishak scoring systems in the training set*

METAVIR system	Ishak system				
	0	1 or 2	3	4 or 5	6
F0	35	0	0	0	0
F1	1	37	0	0	0
F2	0	0	8	0	0
F3	0	0	0	10	0
F4	0	0	0	0	1
Total	36	37	8	10	1

* κ = 0.99

Correlation between the two systems in the validation set

Using the Ishak system, the mean±SD stage of liver fibrosis was 1.60±1.27 (range: 0–5). The mean±SD grade of necro-inflammation was 4.18±2.18 (range: 1–13) in the validation set. Evaluation of the different components of the Ishak grading system showed that the mean±SD score of interface hepatitis was 0.70±0.73 (range: 0–3); the mean±SD score of confluent necrosis was 0.26±0.84 (range: 0–4); the mean±SD score of focal lytic necrosis-apoptosis was 1.51±0.63 (range: 0–3); and the mean±SD score of portal inflammation was 1.70±0.65 (range: 1–3). 

Table 5 shows a comparison of the necro-inflammatory scores between the two systems according to our proposed criteria in the validation set. There was substantial correlation (κ = 0.61) between the two systems using the proposed criteria. 

**Table 5:** Comparison between METAVIR grading system, and the proposed criteria for Ishak grading categorization in the validation set*

METAVIR grading	Ishak grading			
	Minimal	Mild	Moderate	Severe
A0	2	0	0	0
A1	9	39	0	0
A2	0	0	6	0
A3	0	0	0	1
Total	11	39	6	1

* κ = 0.61

We found that most of the discordances between our criteria and METAVIR grades are in the minimal and mild necro-inflammation groups (*e.g.*, A0, and A1 in METAVIR). When we combined minimal and mild necro-inflammation as one group in the Ishak system (*e.g.*, minimal/mild *vs* moderate *vs* severe inflammation), and merged A0, and A1 of METAVIR as one group (*e.g.*, A0/A1 *vs* A2 *vs* A3), the correlation between the two grading systems was perfect (κ = 1.0) in the validation set. 

Then, we analyzed the correlation between the two grading systems using the previously suggested categories [8] for the Ishak system. In the old categorization, the correlation between Ishak and METAVIR grading systems was slight (κ = 0.19). 

Using our criteria, the correlation between staging of METAVIR and staging of Ishak system was excellent (κ = 0.94) in the validation set (Table 6). 

**Table 6:** Comparison between scores for staging of liver fibrosis obtained by METAVIR and Ishak staging systems in the validation set*

METAVIR system	Ishak system				
	0	1 or 2	3	4 or 5	6
F0	9	0	0	0	0
F1	1	35	0	0	0
F2	0	1	7	0	0
F3	0	0	0	4	0
F4	0	0	0	0	0
Total	10	36	7	4	0

* κ = 0.94

Sources of discrepancy in grading of necro-inflammation

To find out the sources of discrepancies, we carefully analyzed the scores given by the pathologists in the training set, and the scores given by the pathologist in the validation set. As expected, the discrepancies were mostly in the minimal/mild necro-inflammation. 

The following finding was the main source of discrepancy. In the training set, the slides with the Ishak score of a0, b0, c1, and d1 were respectively scored in the METAVIR as lobular necrosis: 0, portal inflammation: 1, piecemeal necrosis: 0, and bridging necrosis: 0. This corresponds to A0 in the METAVIR system. However, in the validation set, the slides with similar Ishak score (*e.g.*, a0, b0, c1, and d1) were respectively scored in the METAVIR as lobular necrosis: 1, portal inflammation: 1, piecemeal necrosis: 0, and bridging necrosis: 0. This corresponds to A1 in the METAVIR system [3]. 

## DISCUSSION

The aim of various histological scoring systems for chronic hepatitis is that the same definition of activity be used by all pathologists [3]. The different scoring rules of each system limit our ability to predictably convert scores between them. In this study, we propose criteria which allow more direct comparison of Ishak and METAVIR scores. 

Using our proposed categorization for the Ishak system, correlation of the grading between the two systems was excellent (κ = 0.89) in the training and substantial (κ = 0.61) in the validation set. 

We found that most of the discrepancies observed between our suggested categorization and the validation set were attributed to the discrimination of minimal from mild necro-inflammation. When we merged minimal and mild necro-inflammation as one group, the correlation of the suggested categorization (Table 1) and the validation set was perfect (κ = 1.0). Therefore, our suggested criteria were particularly accurate for discriminating minimal/mild *vs* moderate *vs* severe necro-inflammation.

We found that components “a” (*e.g.*, interface hepatitis) and “b” (*e.g.*, confluent necrosis) of the Ishak system play important role in the correlation with the METAVIR grading system. Furthermore, consistent with the findings of the METAVIR cooperative study group, interface hepatitis (piecemeal necrosis) and lobular necrosis are more important for the grading of METAVIR system [3].

In this study, we compared the two grading systems based on categorical rather than numerical data. In an earlier comparison between the two systems, the total grading of the Ishak was compared with the METAVIR grading system [8]. However, since each component of the Ishak system differs from other components in terms of scale and importance, individual components of the Ishak grading system should be individually analyzed [9]. Indeed, we found a poor correlation between the two grading systems (κ = 0.18) when we compared the two systems according to the numerical data.

Stages 1 and 2 of the Ishak system represent mild fibrosis without bridging. These scores were compatible with F1 of METAVIR system. Stages 4 and 5 of the Ishak system represent advanced bridging fibrosis and/or the beginning of nodule formation. These scores correspond to F3 in the METAVIR system. According to our proposed categorization (Table 2), correlation between the two systems was almost perfect (κ of 0.99 in the training, and 0.94 in the validation set).

We also determined inter-observer variability of each scoring system in the training set. We found an excellent agreement between the two pathologists in both Ishak and METAVIR scoring systems. In another study of inter-observer variability for the Ishak system a moderate to good agreement was reported [10]. The excellent inter-observer agreement found in our study may be explained by the high level of the expertise of the two pathologists. Furthermore, the percentage of agreement is dependent on the number of observers. In conclusion, we found that either scoring systems could be applied for grading and staging of chronic liver diseases. Categorization of Ishak scores allowed accurate translation to the corresponding scores of the METAVIR system.
